# Regulation and functional role of the electron transport chain supercomplexes

**DOI:** 10.1042/BST20210460

**Published:** 2021-11-08

**Authors:** Sara Cogliati, Jose Luis Cabrera-Alarcón, Jose Antonio Enriquez

**Affiliations:** 1 Centro Nacional de Investigaciones Cardiovasculares (CNIC), Madrid, Spain; 2 Centro de Biología Molecular Severo Ochoa (CBMSO), Consejo Superior de Investigaciones Científicas-Universidad Autónoma de Madrid (CSIC-UAM), Madrid, Spain; 3 Centro de Investigación Biomédica en Red Fragilidad y Envejecimiento Saludable (CIBERFES), Madrid, Spain

**Keywords:** electon transport chain, N-respirasome, OXPHOS, Q-respirsome, supercomplexes

## Abstract

Mitochondria are one of the most exhaustively investigated organelles in the cell and most attention has been paid to the components of the mitochondrial electron transport chain (ETC) in the last 100 years. The ETC collects electrons from NADH or FADH_2_ and transfers them through a series of electron carriers within multiprotein respiratory complexes (complex I to IV) to oxygen, therefore generating an electrochemical gradient that can be used by the F_1_-F_0_-ATP synthase (also named complex V) in the mitochondrial inner membrane to synthesize ATP. The organization and function of the ETC is a continuous source of surprises. One of the latest is the discovery that the respiratory complexes can assemble to form a variety of larger structures called super-complexes (SCs). This opened an unexpected level of complexity in this well-known and fundamental biological process. This review will focus on the current evidence for the formation of different SCs and will explore how they modulate the ETC organization according to the metabolic state. Since the field is rapidly growing, we also comment on the experimental techniques used to describe these SC and hope that this overview may inspire new technologies that will help to advance the field.

## Introduction

Mitochondria are a major hub of metabolism and cell signaling since they coordinate the catabolic and anabolic reactions, impact on calcium and ROS signaling, modulate epigenetic regulation and nutrient sensing and determine the execution of cell death programs [1].

**Figure 1.**
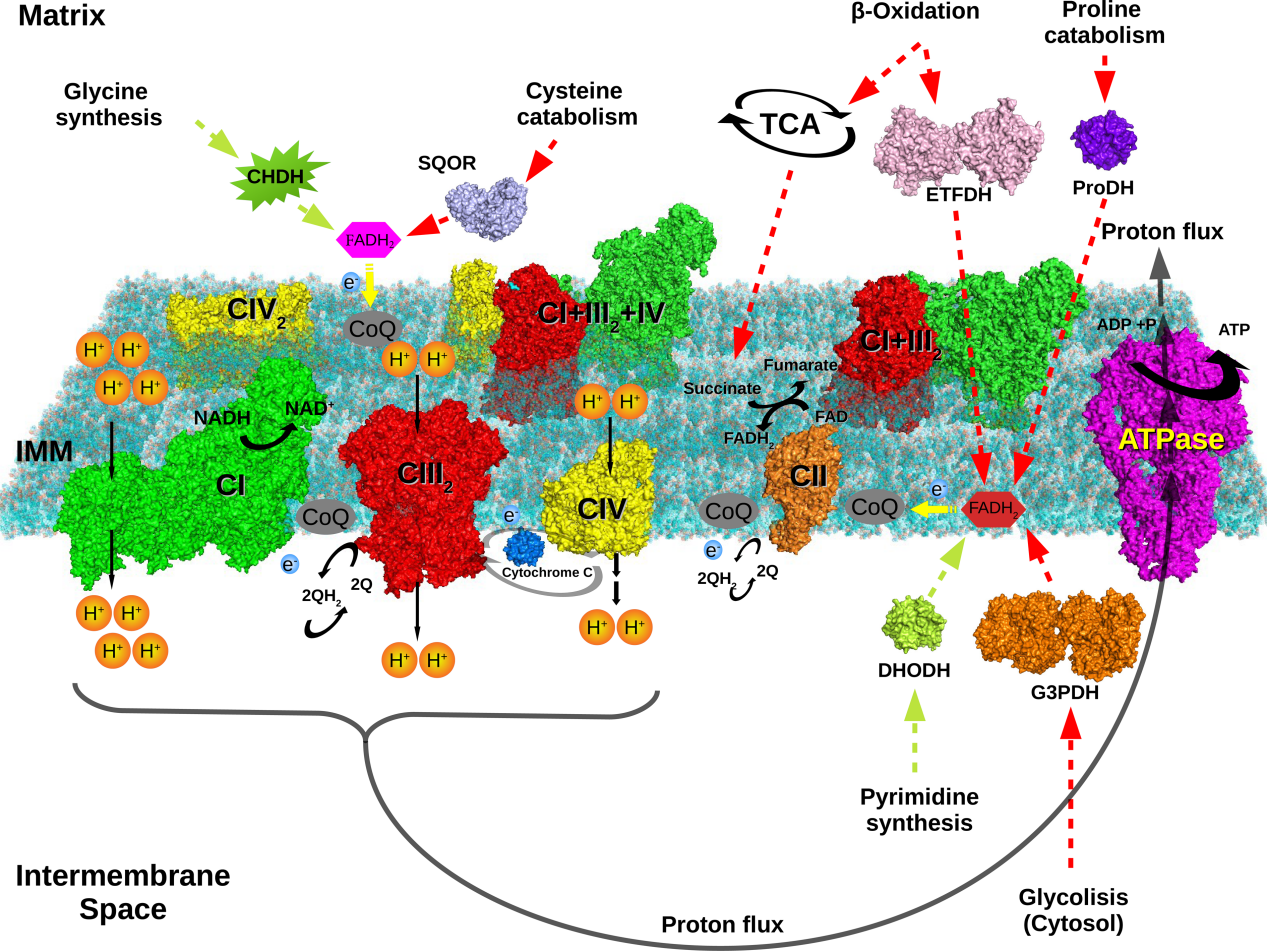
Mitochondrial ETC is the central hub of cellular bioenergetics. Anabolic (amino acid metabolism and nucleotide biosynthesis) and catabolic reactions (glycolysis, TCA cycle, β-oxidation and amino acid oxidation) release electrons stored as FADH or NADH to the ETC that channel them to CoQ. In the picture is outlined the electron flux from the different enzymes of metabolic pathways to the complexes and super-complexes of the ETC. FADH_2_ dependent enzymes attached to it, while NADH generated by a variety of metabolic process navigate to respiratory CI to be oxidized. Anabolic pathways are shown in green and catabolic pathways in red. The following pdb structures were used to develop this figure: 6idj (DHODH, Homo sapiens), 5m42 (proDH, Thermus thermophilus), 6iq6 (G3PDH, Homo sapiens), 6oi5 (SQOR, Homo sapiens), 2gmh (ETFDH, Sus scrofa), CI (5xtd, Homo sapiens), CII (4ytp, Sus scrofa), CIII2 (5xte, Homo sapiens), CIV (5z62, Homo sapiens), CIV2 (1occ, Bos taurus), ATPase (6tt7, Ovis aries), Tight N-respirosome (5j4z, Ovis aries) and lipidic bilayer (2mlr).

The mitochondrial electron transport chain (ETC) is localized in the mitochondrial cristae [2]. It uses the electron transport to generate a proton gradient that is coupled to ATP synthesis by the F_1_-F_O_ATP synthase. The overall process is known as the oxidative phosphorylation (OXPHOS). All the OXPHOS complexes are multiproteic transmembrane structures ranging from the smallest, CII with a mass of ≈140 kDa in mammals and made of four proteins, to the biggest CI made in mammals from 44 unique proteins, being one of them present twice, and with a molecular mass higher than 900 kDa. CIII (11 proteins) and CIV (14 proteins) weigh ≈245 kDa and ≈217 kDa, respectively. The major source of NADH and FADH_2_ reduced equivalents that fed the ETC are generated within the mitochondria at the TCA cycle and β-oxidation, although additional anabolic and catabolic pathways also contribute to both pools. (Figure 1) The transfer of electrons between complexes occurs thought the so-called mobile carriers CoenzymeQ (CoQ) and cytocrome *c* (cyto *c*).

**Figure 2.**
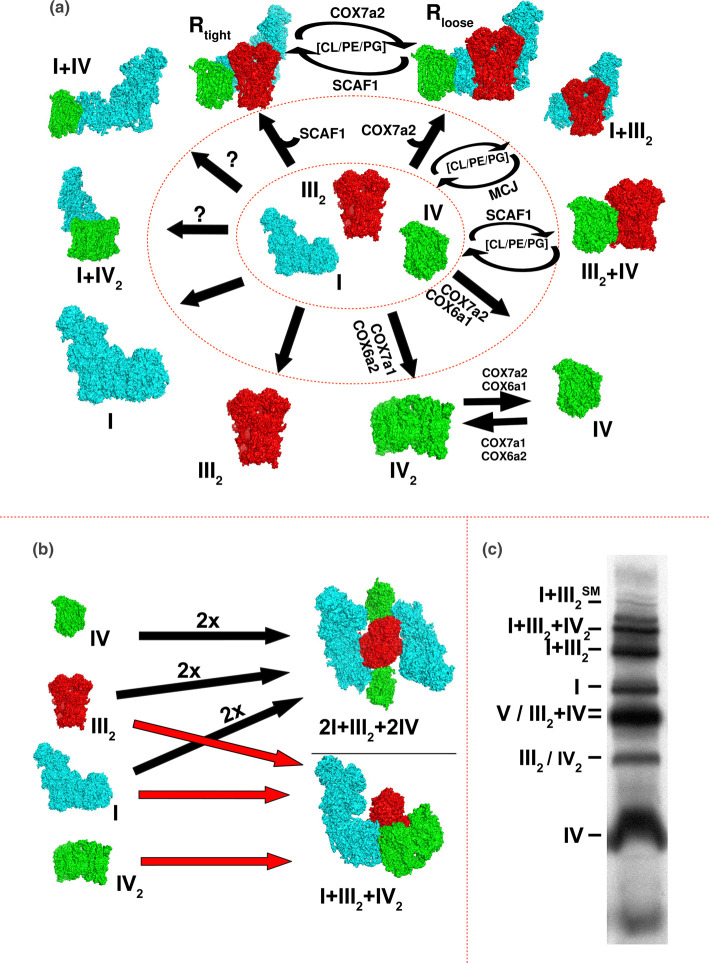
Respiratory complexes and SCs composition. (**a**) Respiratory super-complexes are formed by different composition of complexes. Key factors are the expression of different subunit isoforms important for CIV dimerization (COX7A2, COX7A1, COX6A1, COX6A2), the assembly factors SCAF1 (important for CIII and IV interaction) and MCJ (inhibitor of CI and CIII assembly), MIM lipid composition (CL: cardiolipin, PE: phosphatidylethanolamine, PG: plasmalogen). (**b**) Respirasome alternative ternary interactions. N-respirasome may include a single copy of CIV2 instead of CIV. Moreover, the stoichiometry of megacomplex (2I + III_2_ + 2IV) is represented. The following pdb structures were used to develop this figure: CI (5xtd, Homo sapiens), CIII2 (5xte, Homo sapiens), CIV (5z62, Homo sapiens), CIV2 (1occ, Bos taurus), Loose N-respirasome (5j7y, Ovis aries), Tight N-respirasome (5j4z, Ovis aries) and Megacomplex (Homo sapiens, Bos taurus). **c)** BN-PAGE of 2 h [^35^S]-methionine pulse labeled mtDNA encoded proteins wild-type mouse embryonic fibroblasts from and harvested after 24 h of chase.

The organization of the ETC has been the focus of a vibrant debate for more than 70 years. It was caught between two fires: *the solid vs fluid model* (exhaustive reviewed elsewhere [2,3]). The first considered all the mETC components packed in a unique functional unit [4,5], while the second conceives each respiratory complexes randomly distributed as single and isolated structure, connected by CoQ and cyt *c* [6]. The fluid model become universally accepted, it is considered that CIII is present always in dimeric form (≈500 kDa), while the CI and CII complexes are monomers and CIV can appear either as a monomer or as a dimer. Then, the transference of electrons between complexes occurs by the diffusion of the carriers CoQ and cyt *c*. In 2000, a study using Blue Native Gel Electrophoresis (BN-PAGE) demonstrated that complexes can interact and form large structures called super-complexes (SCs) [7] recovering the idea of the solid model. Then it was proposed the existence of two types of SCs or respirasomes I + III_2_ + IV_4_ (NADH-respirasome or N-respirasome) and III_2_IV_4_ (CoQ respirasome or Q-respirasome) in a proportion of 2 N-respirasomes per Q-respirasome, to fit the overall 1 : 3 : 6 stoichiometries of complexes I : III : IV [7]. It was also postulated that those structures could be combined to form ‘respiratory strings’ as the physical concatenation of N- and Q-respirasomes forming long chains in the mitochondrial inner membrane [8–10]. The remaining observed associations like the abundant SC I + III_2_ were considered broken parts of bigger entities. However, the existence of respiratory strings could not be experimentally supported and none of the hypothesized stoichiometry for the N- or Q-respirasomes has ever been documented. Instead, molecular [11–13] and structural evidence [14–17] supports the existence, of the N-respirasome as SC I + III_2_ + IV_1–2_ (Figure 2a), that may further associate as megacomplex 2I + III_2_ + 2IV [18] (Figure 2b). The Q-respirasome is observed as SC III_2_ + IV and SC III_2_ + IV_2_ [11–13] (Figure 2a).

With quantitative variations between different sources of mitochondria, the variety of super assemblies between respiratory complexes observed by BN-PAGE is constant (Figure 2c). Recently the scientific debate has focused on the organization of C_O_Q and cyto *c* pools [19], the impact of SC assembly for the activity of the complexes, the molecular mechanisms that drive the super assembly [2,20] and the bioenergetics and physiological role of SCs.

## How many super-complexes exist?

The debate about the ETC organization [2] ended in 2008 with the proposal of the *plasticity model*, that considers the coexistence of complexes and SCs [21,22]. Thanks to a combination of genetic ablation of individual respiratory complexes and BN-PAGE, it was possible to identify SCs dependent on molecular interactions and discriminate them from spurious co-migration and breaking part of bigger structures [2,21,22]. In this model, CI, CII and CIV can be seen a monomer, CIII is always a dimer while and CIV can be also found as dimer. Further CI, CIII and CIV form pairs or trios with other respiratory complexes in significant proportions Thus, besides the dimers of CIII and CIV and the N- and Q- respirasomes, additional binary associations to form SC I + III_2_, SC I + IV and SC I + IV_2_, whose function is uncertain, have been described [13] (Figure 2). Of note, the proportion of respiratory complexes appearing in free form or associated varies depending on the species [12,23–28], cell type [13,29–32], and physiological situation [24,34,35,28,33].

The first estimation of the distribution of complexes between free form and SCs was provided for bovine heart [36]. Thus, CI was mainly found in SCs with ≈15% in the free form [36], a proportion that is even lower in human cell lines [37] and human skeletal muscle [24]. For that reason, the existence of CI as an individual isolated complex was considered as artefact due to the solubilization of mitochondrial membrane with digitonin, a fundamental step for BN-PAGE analysis [7,38]. However, in rodents, bovine, zebrafish, Drosophila, fungus and plants, the persistent presence of free CI is observed [7,12,21,39–41] and can be modulated by different physiological stimuli [34,42]. Together with that, a careful kinetic analysis of CI assembly in human cells found that CI is fully assembled individually and quickly stabilized by super assembly [37]. All this data argues in favor of the existence of free CI.

Regarding bovine heart, more than 40% CIII was found as III_2_ while more than 80% of CIV was found as a monomer [36]. Mouse and rat brain mitochondria show very low levels of free CI and a higher proportion of the respirasome compared with other tissues. In most cases, CIV appears as a monomer with a small fraction as dimers or associated to SCs, more abundant in heart and skeletal muscle than liver. Brown adipose tissue is characterized by high levels of the Q-respirasome (III_2_ + IV) [43]. Now, the consensus is that BN-PAGE has cataloged two major classes of SCs in animal cells: binary (SC I + III_2_ and III_2_ + IV) and ternary (SC I + III_2_ + IV) for which there is experimental evidence that co-migration is due to true interaction (Figure 2c), since the elimination of one complex modifies the migration of other(s) [21]. In addition, the significant increase in resolution allowed by cryo-slicing BN-PAGE samples combined with Mass Spectrometry analysis (named in general ‘complexome analysis’) led to the proposal of two novel binary SCs: I + IV and III_2_ + IV_2_ [11], and recently, similar SCs were described also in zebrafish [12] and in mice [13]. The ternary structures were named N-respirasomes because they contain the three complexes that could allow a full respiratory chain [7], and their capacity to respire from NADH was later confirmed [21]. Besides the more commonly reported SCs, a frequently observed association between I + III_2_ of very high molecular weight can be observed that may be compatible with 2(I + III_2_) or the consequence of interaction with undetermined components [21]. In addition, a gigantic structure of ring-shape named megacomplex 2I + III_2_ + 2IV has also been reported [18]. The isolation of this megacomplex from HEK293T cells calls for caution regarding the relevance of this megacomplex in more physiological contexts (Figure 2a,b). Nowadays, it is still not completely clear whether CII could interact with other respiratory complexes. Indeed, in mammalian, it is mainly found in free form and barely co-migrating to other complexes in a BN-PAGE especially if the solubilization conditions are not stringent [21]. Another hypothesis, from *in silico* modeling, suggests that CII can fit into the ring of the megacomplex but experimental evidence is lacking [18]. Even if not part of any SC, CII has a role in the modulation of SC assembly under oxygen concentrations and NADH/FADH_2_ ratio [34]. On contrary, in pea shoot, CII has been described associated to a megacomplex II_x_III_y_IV_z_ [44] whose function and role are still unknown. Although this is out of the scope of this review the observation of CII migrating in high molecular positions in BN-PAGE may be more related to the postulated formation of associations with other enzymes of the TCA cycle called TCA metabolon [45].

The detection of co-migration of several respiratory complexes in the BN-PAGE gel by immunoblot or in gel activity is insufficient to demonstrate that they physically interact [21]. In this regard, the co-migration of I + IV_2_ and I + III_2_ may be misunderstood as respirasome [46]. Visualization and dynamically tracking the ETC organization *in situ* have started to show results [47–51], but may still need improvement to be able to track endogenous levels of SCs.

## Mechanisms and factors of SCs assembly

Experiments of genetic modulation of complex subunits, the use of detergent-free methods and the resolution of SCs structures by cryo-electron microscopy (cryo-EM) together with the discovery that SCs assembly is a genetic regulated process, definitively prove the existence and relevance of SCs.

In 2012–2013 several groups proposed candidate proteins responsible for the assembly between CIII and CIV [42,52–54]. They were named Rcf1 and Rcf2 in yeast [52–54] and HIGD1 and HIGD2 in mammals. Rcf1 and Rcf2 are members of the hypoxia-induced gene (domain) 1 Hig1 family, and later studies demonstrated that they are not required for SC assembly rather than for the stability, activity [55,56] and assembly of CIV [57,58]. Therefore, the only bona-fide SC assembly factor required for III_2_ and IV interaction is the subunit COX7A2L renamed as Super Complexe Assembly Factor 1 (SCAF1), discovered in 2013 [42]. Its absence does not affect any aspect of either individual CIV or CIII assembly, stability, or function [2] but affects the N- and Q- respirasome structures.

An unexpected finding, that later was pivotal for unveiling SCAF1 role, revealed that the more commonly used inbred mouse strain (C57BL/6 and their sub-strains) harbors an in-frame micro-deletion that renders SCAF1 non-functional [42,59,60]. Therefore, this strain lacks the Q-respirasome. The role of SCAF1 in the formation of N-respirasome turns to be more complex but it was recently solved [13,61]. Detailed complexome analysis showed that two different N-respirasomes can be formed with different subunit composition of CIV, ether with SCAF1 or with COX7A2 [13,61]. The N-respirasome with SCAF1 has a structural attachment between III_2_ + IV while the one with COX7A2 lacks physical interaction between complexes III and IV [13]. Both versions of the N-respirasome are functionally distinct, the presence of SCAF1 increases NADH-dependent respiration and reduces reactive oxygen species (ROS) production [13] resulting in better response to severe fasting and exercise in mice and zebrafish [12,13]. On the contrary, SCAF1 depletion in human embryonic kidney cells (HEK293T) does not apparently affect OXPHOS performance [61], probably because they barely express SCAF1 in basal conditions and almost lacks naturally SCAF1 containing respirasome [61].

The available cryo-EM structures of the N-respirasome were obtained from a single tissue (heart) and lack of sufficient resolution to determine which subunit of the COX7A family (COX7A1, COX7A2 or COX7A2L/SCAF1) is present in the structure [14–17]. Proteomic analysis allows to discard the presence of COX7A1 since is preferentially found in complex for dimers in heart mitochondria [29] (Figure 2a). Therefore, it is uncertain which of the N-respirasomes (COX7A2 containing, SCAF1 containing or a mix of both) was analyzed [14–17]. Further improvement in resolution is required to address this point. Cryo-EM analysis also suggests the existence of two N-respirasomes with different degree of interaction of III_2_ and IV, the tight N-respirasome with contact between III_2_ and IV, and the loose N-respirasome and where no physical contact between III_2_ and IV is observed [14,16]. The gradual transformation of the tight form into the loose form in solution was confusing. The dynamicity of the III_2_ + IV interaction in the N-respirasome can justify also why the different areas of the respirasome have variable resolution. Indeed, CI has always the best definition, while CIV has the worst probably due to its ability to dynamically adopt alternative positions. A puzzling consistent observation about the N-respirasomes is that in BN-PAGE, they migrate as a series of closely bands that may correspond to apparently different molecular weights. One old speculative interpretation claims that each band represents the addition of one copy of CIV, from 1 to 4 [36], but recently it has been found that regardless of the migration position of the N-respirasome, the stoichiometry between three complexes was substantially constant in the form of I_1_ + III_2_ + IV_1_ with only a minority amount of I_1_ + III_2_ + IV_2_ [13]. The reason for different migration remains unclear but it has been proposed that the COX7A2 containing N-respirasome migrate slightly faster than the SCAF1 containing N-respirasome suggesting that not only the mass, but the shape of the structures contributes significantly to their migration in the BN-PAGE [61].

Regarding the Q-respirasome a recent cryo-EM structures from mouse and ovine heart has been reported [62]. This structures fully confirm the role of SCAF1 in holding together both complexes and the SCAF1 domains responsible for the interaction with CIII and CIV derived from biochemical and mutagenesis analysis [29].

SC I + III_2_ [14,16,63], the megacomplex 2I + III_2_ + 2IV and the interaction between III_2_ and IV in the N-respirasome could not be fully resolved due to the lower resolution of complex IV in those structures. Interestingly, it was observed *in vitro* in a controlled set up the progressive separation of the III_2_ + IV in the Q-respirasome and the detachment of IV from the N-respirasome, reminiscent of the tight to loose transformation of the N-respirasome observed by cryo-EM [14,16]. It could be determined that it is caused by the calpain proteolytic cleavage of SCAF1.

**Figure 3.**
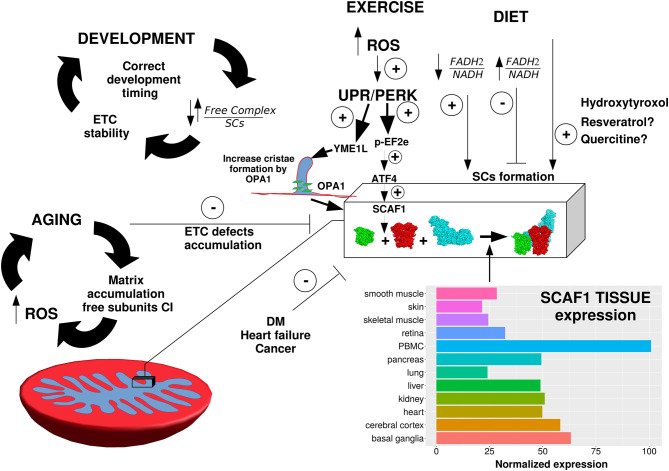
Metabolic adaptation of super-complexes. Super-complexes formation undergoes to adaptation upon different metabolic conditions. During development, the formation of super-complexes follows a genetically coordinated timing. Exercise improves super-complexes formation probably trough a ROS/UPR/PERK mediated pathway. Endoplasmic reticulum stress response triggers SCs assembly through the PERK axis that activates both SCAF1 expression and cristae formation**.** The accumulative damage may be due to ROS increasing is responsible of super-complexes damage during aging and probably also in diabetes mellitus (DM) and heart failure. The massive dependency on anabolic reactions of cancer cells could be responsible of the super-complexes increase. Diet and in particular the ratio NADH/FADH_2_ modulate super-complexes distribution through RET. The different expression levels of SCAF1 in different tissues could be responsible of the variability in CIII_2_ + IV and CI + III_2_ + IV amount. Expression data obtained from Human Protein Atlas available from http://www.proteinatlas.org. The following pdb structures were used to develop this figure: CI (5xtd, Homo sapiens), CIII2 (5xte, Homo sapiens) and CIV (5z62, Homo sapiens).

The mechanism of I and III_2_ super assembly is mostly unknown, but several proteomic analyses strongly support that this interaction does not need any assembly factor [11,29,37]. Noteworthy, a protein called MCJ/DnaJC15 that interacts with complex I, reducing its activity and preventing its association into SC, has been proposed as negative regulator [64]. Several studies indicate that the lipid environment, specifically cardiolipin, determines the super assembly between CI and CIII [65–68]. In agreement with this, Tafazzin mutant, which affects cardiolipin synthesis, manifest the loss of SCs containing CI [67,69,70]. Also, mutations of Prohibitin [71] and Stomatin [72,73], impair the formation of CI and CIII interactions. The formation of SCs requires not only a favorable lipid environment and the participation of specific modulators, but also a well-defined cristae structure [74]. This was revealed by the loss of the SCs upon ablation of OPA1, a fundamental protein that regulates mitochondrial cristae formation, stability, and dynamics [75]. This multifaceted interaction between specific factors and the environment was illustrated by the deciphering of the mechanisms of SC formation induction by ER stress [33]. This induction required the ATF4 mediated activation of SCAF1 expression and the independent and parallel increasing of cristae density [33] (Figure 3).

## Functional relevance of SCs

The findings that CI stability in mammals is impaired by the absence of CIII [76,77] and CIV or cyt *c* [78] led to the proposal that the SCs either stabilize the respiratory complexes [27,76,77] or serve as a platform where CI is assembled [38,79,80]. Recently, a series of experiments suggested that in the absence of either CIII or CIV, CI is quickly degraded due to the activation of retrograde electron transfer (RET) when the proportion of reduced CoQ is abnormally elevated [34] and it is rescued by the RET prevention [34] or the expression of an alternative oxidase capable of re-oxidizing CoQH_2_ (bypassing the role of CIII, cyt *c* and CIV together) [34]. Therefore, the stability of CI seems to be more dependent on the prevention of RET rather than on the actual formation of super-complexes. An additional and complementary explanation of the CI dependency on CIII was suggested by using human cell lines defective in CIII assembly [38]. These results suggested that the last step in the assembly in CI, the incorporation of the N-module (responsible for the NADH dehydrogenase activity) requires the previous interaction of the partially assembled CI with CIII [38]. Therefore, it is proposed that, in this cellular model, CI finish its assembly after the interaction with III_2_ [38,79].

In isolated I + III_2_, super assembly of CI and CIII impact on the capacity of CI to oxidize NADH [63] and diminishes the accessibility of external decylubiquinone (DQ) to CI [63], suggesting that the assembly of CI into SCs may partially protect it against RET and subsequently prevent its degradation. Along this line, the activation of SCs formation by the protein kinase R (PKR)-like endoplasmic reticulum kinase (PERK), stabilizes CI and therefore supports the growth in galactose of cells harboring missense mutations [33]. In conclusion, the formation of SCs stabilizes CI, but this interaction does not seem to be mandatory to allow fully assembled and functional CI in all physiological scenarios. Thus, CI can be fully assembled independently of its interaction with CIII [13,34,37,38,76] or being its N-module added after super assembled with CIII [38,79].

A second functional role proposed for SC is the organization of the electrons flux to enhance respiratory activity preventing electron traffic jams [34,42,81], and minimize ROS production [13,32,82]. CI and CIII containing SCs harbor CoQ in their structure that is sufficient to trigger the oxidation of NADH to cyt *c* [38]. Two questions need to be addressed: (i) is the CoQH_2_, generated by CI, released to the membrane milieu and mixed and equilibrated with the CoQH_2_ generated by other enzymes (like CII) before being oxidized by CIII? (ii) is the CoQH_2_ generated outside the SCs capable to be oxidized by CIII assembled to SCs together with CI. The two questions refer to the existence or not of metabolic channeling between complex I and III_2_ in SCs. Metabolic channeling can be enforced by building permanently sealed protein tunnels within the SCs connecting the different reaction centres or by the differential partitioning of the intermediate metabolites that prevent its free diffusion. Structural analysis from different groups demonstrates that the cavity that contain the CoQ binding sites of CI and CIII in the I + III_2_ SC is not sealed, and that CoQ would be able to diffuse out of the SC [14–16,63]. However, the kinetic of the flux of electrons from CII to CIII [42] or from NADH to two different AOX enzymes [13,83] is negatively affected by the formation or activity of SCs I + III_2._ These results are in discrepancy with those published by Fedor et al. [84]. The discrepancy may relay on the very different experimental conditions used. Thus, the former was observed by *in vivo* genetic manipulation of the I + III_2_ proportion and by *in vivo* expression of AOX [13,42,83]. On the other hand, Fedor et al. [84] used heart derived submitochondrial particles to whom bacterial expressed recombinant AOX was added *in vitro*. The same question applies to cyt *c* in the context of CIII and CIV containing SCs. In this case all reports are coincident in showing that electron transfer between III_2_ and IV within the mammalian Q-respirasome is more efficient than between free III_2_ and free IV [62] confirming in this way the postulated of the plasticity model [22,42]. Interestingly, this view is in full agreement with the observations of the role of the yeast respirasome [85,86]. For a detailed discussion on the functional segmentation of CoQ and cyt *c* see [19,87].

Several mechanisms can account for impact of SCs in the partial segmentation of the CoQ and cyt *c* pools. In the case of CoQ the lipid milieu surrounding the SC and the rest of the membrane can favor its turnover within the SC by partitioning, while cyt *c* can be retained in the SC by electrostatic forces [62,85,86]. Additionally, in both cases the proximity between the reaction centres caused by super assembly can favor its turnover within the SC.

## SCs under different metabolic conditions

It is known but often neglected that the electron equivalents generated in catabolism that fuel the mitochondrial electron transport chain may have two different flavors: the soluble NADH, that would be oxidized through CI, or FADH_2_ that delivers electrons to the mitochondrial electron transport chain bypassing CI [81,88]. Very important, different substrates generate different proportions of FADH_2_/NADH (F/N) electrons, and this ratio plays a fundamental role in ROS generation by mitochondria [81,88]. In particular, the higher the F/N ratio the higher the CoQH_2_/CoQ ratio and this could facilitate, in conjunction with high membrane potential, the induction of RET. This phenomenon reduces, both in cell lines [34] and mouse liver [42], the proportion of CI assembled with CIII therefore increasing the fraction of CIII available for FADH_2_ enzymes to favor fatty acids oxidation [34]. This suggests that the dynamic modulation of SCs’ proportion is a mechanism to efficiently adapt to the available carbon sources (Figure 3). Thus, in cultured cells the proportion of CI in free form vs SCs is higher when mitochondria oxidize pyruvate than when they oxidize fatty acid [34]. Likewise, liver mitochondria from overnight fasted male mice reduce the proportion of CI containing SCs [42].

The observation that SC formation is modified in response to ER-stress [33] and the impact of calcium and sodium in the regulation of MIM fluidity and OXPHOS activity [89] reinforce the role of the super assembly in adapting the metabolic response of the mitochondria. In this context, mitochondrial shaping proteins are important not only in the organelle dynamics but participate in regulating the architecture of the cristae to allow SCs formation [33,74,75]. Under this perspective, it is expected that the organization in SCs would respond to metabolic differences induced by cell type, physiological changes and environmental cues that impacts on metabolism. This assumption is corroborated of growing body of evidence where SCs formation is enhanced or reduced to face different metabolic requirements. A great example is physical activity that increases the assembly of SCs in both humans and rats [24,35]. A more causative link comes from the demonstration that wild-type SCAF1 is necessary to achieve maximum exercise performance both in mice [13] and zebrafish [12]. Without a description of a precise molecular mechanism, we can only speculate that it can be induced by the activation of the PERK-ATF4 axis [33] and the concomitant expression of mitochondrial remodeling enzymes like OPA1 [90] (Figure 3).

Heart failure, and ischemia/reperfusion are characterized by a decrease in respirasome proportions and OXPHOS capacity [91–93] leading to the hypothesis that targeting SC formation could be a promising therapeutical approach for these pathologies. The brain offers an extraordinary example of metabolic cooperation between cells. The high energetic demand of neurons is satisfied by an efficient cooperation between astrocytes and neurons [94]. The former is glycolytic and generates and releases lactate that is taken by neurons to perform oxidative phosphorylation. Thus, the proportion of free vs super assembled CI in mice astrocytes is higher than in neurons [32]. This is accompanied by lower respiration activity and more ROS formation in astrocytes, providing a functional link between the organization of SCs and mitochondrial metabolism [32].

SCs organization is rearranged during immune response in macrophages. Mouse macrophages activated by live *E. coli* decrease the SCs containing CI and increase free CIII. Consequently, there is an increase in the activity of CII and glycerol-3-phosphate dehydrogenase that allows the use of FADH_2_ as substrate [30]. Along the same line, it was reported that an incremental increase in CII activity in LPS-activated mouse macrophages together with a decrease in the NAD^+^/NADH ratio activated RET, thereby producing ROS [95]. This mechanism potentiates the inflammatory state. While the number of studies on immune metabolism and SCs association are growing, little is known about the role of mitochondria in development and if and how the organization of SCs affects organogenesis is still an open question. Recently, a comprehensive analysis in zebrafish demonstrated that the SCs appear at early stages of embryonic development, and their number and associations increase progressively [96]. Few studies in mammals showed that SCs formation increases during adipogenic differentiation of human mesenchymal stem cells and, in embryonic mouse heart, SC formation starts at E11.5, concomitant with a burst in OXPHOS activity [96]. Further studies corroborate that mouse neonatal cardiomyocytes assemble less SCs than adult cells, supporting the idea that specific metabolic requirements induce the formation of SCs [29]. Interestingly, in cardiomyocytes lacking the transcriptional repressor CTCF, SCs formation is blunted accompanied with a disruption of the cardiac developmental program [96]. On the contrary, with age, SCs decrease at least in the brain [97] and heart [98] (Figure 3) suggesting that SCs formation goes together with the higher energetic demand of the tissues.

Two important pathologies are associated with changes in SCs association: diabetes and cancer. A unique study reported that SCs assembly is reduced in the *rectus abdominalis* muscle of diabetes patients and this correlates with poor mitochondrial function [99], but the precise molecular links and whether the pathological condition can be ameliorated by compensating for the loss of SCs remains unclear. One therapeutic intervention could be a specific diet or the addition to food derived components. An example that shed light on this possibility, is hydroxytyrosol [100], a component of olive oil, that burst the formation of SC in rat muscles. Another example is dietary fatty acids whose consumption modifies the composition of the mitochondrial inner membrane [101–103]. Despite of evidence that they can modulate mitochondrial functions and complexes activities [104–108], there are no studies regarding the modulation of SC assembly by modulation of dietary fatty acids. We dare to anticipate that it is going to be an important field of research with potential applicability [109–112].

Cancer is a complex pathological condition characterized by altered metabolism, genetic mutations, distorted cell cycle, uncontrolled immunomodulatory factors, and disorganized tissue architecture [113]. Nowadays, great effort is made in elucidating the heterogenous metabolic profiles found in cancer together with the dynamic rearrangement during tumor progression to accommodate and adapt to environmental cues [114]. In this complex landscape, mitochondria and more specifically, ETC are critical players [115]. However, the potential relevance of the SCs dynamics has not been systematically investigated. Interestingly, SCAF1/COX7A2L was discovered as a strong estrogen induced gene [116]. Thus, tamoxifen resistance, a selective estrogen receptor modulator widely used to treat breast cancer, has been connected to SC formation. Her2^high^ tumors, characterized by tamoxifen resistance, have more SCs and more CI dependent respiration [117]. Others reported a correlation between reduction in SCs and elevated resistance [118]. In favor of the pro-tumoral effect, it was estimated that expression of SCAF1 is an unfavorable prognosis marker for liver cancer (The human protein atlas project). In agreement with that, it was reported that the deficiency in MCJ/DnaJC15, which negatively regulates SC formation, is associated with multidrug resistance in mouse and human breast cancer [64]. SCAF1 was found overexpressed in clinical breast and endometrial cancer. Thus, it increases the stabilization of SCs together with a modulation of the metabolism of the TCA cycle intermediates that make tumor cells more resistant to hypoxia and this effect is dampened by the silencing of 2-oxoglutarate dehydrogenase complex [119]. These results clearly show that the formation of SCs is strictly related to the modulation of metabolism especially in oestrogen dependent tumors. More studies are needed to understand the molecular mechanisms and the metabolic adaptation in response to modulation of SCs in tumorigenic conditions. In this direction, SCs is also indirectly regulated by p53 through DPYSL4, belonging to the collapsing response mediator family [120]. DPYSL4 localizes with SCs in BN-PAGE suggesting that it may function in assembly or stability of SCs but further studies are needed to understand its mechanism of action and why SCs formation impact tumor growth in a lung-metastasis model [120].

## Perspectives

- After a strong debate on the existence and function of SCs, a growing body of evidence confirm that SCs exist, and are essential elements for metabolism. Therefore, they are key elements in understanding and eventually manipulating metabolism.
- The assembly of SCs optimizes and organizes the flux of electrons, controls ROS production and modulates the activity of complexes. *In vivo* they confer metabolic advantage. However, we are just starting to understand these processes and many critical questions remain to be answered.
- In the future, studies on SCs dynamics, regulation and physiological implications will be pivotal to understand metabolism and develop strategies for therapeutic purposes. Moreover, an improvement of the techniques to track their dynamic composition *in vivo* will be mandatory.

## Abbreviations

BN-PAGE: Blue Native Gel Electrophoresis
ETC: electron transport chain
OXPHOS: oxidative phosphorylation
PERK: (PKR)-like endoplasmic reticulum kinase
RET: retrograde electron transfer
ROS: reactive oxygen species
SCAF1: Super Complexe Assembly Factor 1
SCs: super-complexes
